# Tinaspora Cordifolia

**Published:** 1894-11

**Authors:** D. N. Banerjee


### TINASPORA CORDIFOLIA.

Chart illustrating the first analysis of proving of the new Indian drug Tinaspora Cordifolia, prepared in proof spirit according to G. H. P., by Sir The Commandeur Dr. D. N. Banerjee.

| | FIRST PROVER. 10 gtts. a dose of $\phi$ twice daily. | SECOND PROVER. 3 gtts. of $\phi$ once a day. | THIRD PROVER. One gtt. a dose of $\theta$ twice daily. | FOURTH PROVER. 5 gtts. a dose of $\phi$ twice daily. |
|---|---|---|---|---|
| MIND | | | Depressed, dullness. | |
| HEAD | Pain in the r. occipital region, heaviness of forehead, heaviness of l. temple. | Headache. | Heaviness, aching of the occipital region, r. sided headache. | Headache, heaviness. |
| EYES | Throbbing of the l. upper lid and r. lid. shaking of the r. lower lid and upper lid. | | Burning sensation. | |
| EARS | | | Ditto. | |
| MOUTH | | | Flow of saltish water. | Swelling of the gums. |
| THROAT | | | Burning sensation. | |
| APPETITE | Usual, good. | Good, loss of. | Loss of, good. | Usual, loss of. |
| STOMACH | Wind, nausea. | Aching of the epigastrium, vomiting of watery fluid, retching, swelling, warm. | Nausea, feeling warm | Nausea. |
| ABDOMEN | Aching at last night. | | | |
| RECTUM AND ANUS | Flatulence. | | | |
| STOOL | Soft, sufficient, free. | Hard, free, diarrhœic. | Free, insufficient. | |
| URINARY ORGANS. | Insufficient, free, painful, pressure, foamy. | Free, sufficient, frequent. | Free, insufficient. | Free. |
| CHEST AND SHOULDER. | | Pain in the left ribs, aching of the sternum, throbbing of the left chest. | Burning sensation, stitching pain in the shoulder and back, pain in the chest during expectoration, aching of the sternum. | |
| HEART AND PULSE. | Pulse 75 and respiration 20 in a minute. | Aching and pain in the r. ribs, palpitation, pulse 74 to 84, respiration 15 in a minute. | Throbbing pain in the r. ribs, pulse 75 and respiration 18 in a minute. | Pulse 70 to 95, respiration 23 in a minute. |
| NECK AND BACK. | Pain in the r. neck. | Pain in the neck. | Pain in the r. neck, aching of the back, aching of the r. scapulæ. | |
| SUPERIOR EXTREMITIES. | Profuse sweat of the palms, aching of the hands, burning sensation of the left palm. | | Pain in the r. arm, burning sensation of the fingers, aching of the l. arm, burning sensation of the l. arm, burning sensation of the tip of the r. fingers, aching of the r. wrist, burning sensation of the r. palm. | |
| INFERIOR EXTREMITIES. | Pain in the left groin and hip, profuse sweat of the feet. | Aching of the legs, pain in the r. thigh. | Aching of legs, knees, and thighs. | Burning sensation between the toes |
| SKIN | Profuse sweat of the palms and feet. | | Burning sensation. | Eczema. |
| SLEEP AND DREAMS. | Usual, good. | Deep sleep, feeling sleepy at 1 P. M., disturbed. | Sound, disturbed in the 8th day. | Usual, late rising, dreams, good. |
| FEVER AND CHILLINESS, ETC. | | Pain in the body, with burning sensation. | Burning sensation, pain in the body. | Pain in the body. |
| RESPIRATORY ORGANS. | Cough dry. | Cough. | | Cough. |
| GENERAL | | | Weakness. | |

